# Exercising Physiological Pathways: The Impact of Exercise on Metabolic Aging, Cellular Aging, and Inflammatory Biomarkers in All‐Cause Dementia

**DOI:** 10.1002/alz.088007

**Published:** 2025-01-09

**Authors:** Mitchell T Hanson, Yanbin Dong, Haidong Zhu, Andre Soares, Colleen Hergott, Jennifer L Waller, Lufei Young, Dawnchelle Robinson‐Johnson, Richard Sams, Mark Hamrick, Deborah A Jehu

**Affiliations:** ^1^ Medical College of Georgia, Augusta, GA USA; ^2^ Georgia Prevention Institute, Augusta, GA USA; ^3^ Augusta University, Augusta, GA USA; ^4^ University of North Carolina, Charlotte, NC USA; ^5^ Claiborne Assisted Living Facility, Augusta, GA USA; ^6^ Georgia War Veterans Nursing Home, Augusta, GA USA; ^7^ Institute of Public and Preventative Health, Augusta, GA USA

## Abstract

**Background:**

Physiological changes, including metabolic and cellular aging, as well as increased inflammation, occur in people living with dementia (PWD). While there is existing evidence in other populations suggesting that exercise may improve physiological outcomes, their impact in PWD remains unclear. This randomized controlled trial (RCT) aimed to assess the effects of exercise on serum levels of metabolic aging, cellular aging, and inflammatory blood biomarkers relative to usual care alone in PWD.

**Method:**

This pilot RCT involved n=42 PWD (exercise n=21; usual care n=21) in one nursing home and one assisted living facility (NCT05488951). The adapted Otago Exercise Program involved 30 minutes of tailored lower body strength and balance exercises and 30 minutes of walking 3x/week for 6 months. We drew fasted blood at baseline and 6 months. Blood samples were stored at ‐80°F and analyzed for metabolic aging (kynurenine), cellular aging (telomere length), and inflammatory biomarkers (interleukin‐6, IL‐1b, interferon alpha2, IFNg, tumor necrosis factor a, chemokine ligand 2, IL‐8, IL‐10, IL‐12p70, IL‐17A, IL‐18, IL‐23, IL‐33). Generalized mixed models were used for intent‐to‐treat (n=42) and per protocol analyses (usual care: n=21; n=9 exercisers with ≥2/3 adherence), controlling for age, race, sex, and the Montreal Cognitive Assessment.

**Result:**

The intent‐to‐treat analysis revealed no differences in physiological biomarkers between groups. The per protocol analysis revealed a trend for reduced inflammation in IL‐1b (exercise: 9.73 to 6.16 pg/mL; usual care:10.51 to 15.46 pg/mL; p=0.09) and IL‐8 (exercise: 39.34 to 25.92 pg/mL; usual care:78.99 to 93.04 pg/mL; p=0.09). Additionally, the control group increased telomere length compared to the exercise group (exercise:8.0 to 7.9 kb; usual care:7.9 to 8.7 kb; p=0.01).

**Conclusions:**

The trend for exercise reducing inflammation in the per protocol analysis suggests that a greater amount of exercise (i.e., ≥2/3 exercise adherence) may be necessary to reduce inflammation in PWD. The nuanced relationship between exercise reducing cellular aging in the usual care group requires further exploration, as we had a small sample size in our per protocol analysis. Our pilot findings may inform a larger RCT to determine a potential interplay between exercise and physiological biomarkers in PWD.

